# Note-Level Phenotyping of Multiple-Sclerosis Notes by a Large Language Model Achieves near Human-Level Agreement

**DOI:** 10.3390/jcm15114092

**Published:** 2026-05-25

**Authors:** Daniel B. Hier, Pavankumar Y. Srinivasula, Michael D. Carrithers

**Affiliations:** 1 College of Medicine, University of Illinois at Chicago, Chicago, IL 60612, USA; mcar1@uic.edu; 2 College of Liberal Arts and Sciences, University of Illinois at Chicago, Chicago, IL 60607, USA; psrin4@uic.edu

**Keywords:** multiple sclerosis, phenotype, clinical text, large language models, natural language processing, inter-rater agreement, phenotyping

## Abstract

**Background/Objectives**: Clinical phenotyping from narrative electronic health records (EHRs) often relies on multi-stage pipelines involving span-level extraction, ontology mapping, and aggregation. Large language models (LLMs) may enable direct document-level abstraction of clinically meaningful phenotype features from complete notes. We evaluated whether GPT-5.2 could approximate human annotation for note-level multiple sclerosis (MS) phenotyping and compared its performance with human annotators, a locally run open-source LLM, HPO-based extraction tools, and a supervised clinical transformer encoder. **Methods**: We analyzed 100 de-identified MS neurology progress notes from a single academic medical center. Each note was annotated for the presence or absence of 17 predefined neurological phenotype categories. Two human annotators independently labeled all notes using a multi-label note-level framework in Prodigy, and disagreements were adjudicated to create a reference annotation set. GPT-5.2 was evaluated in a zero-shot setting using structured JSON output. Comparator methods included Llama-3.1 8B, Doc2Hpo, ClinPhen, PhenoSnap, and BioClinical ModernBERT. Performance was assessed using agreement, precision, recall, F1, Matthews correlation coefficient, and false-positive and false-negative assignments per note. **Results**: Human–human agreement was generally high, although lower for rare or ambiguously documented features. GPT-5.2 achieved the strongest automated performance, with macro-precision 0.734, macro-recall 0.921, macro-F1 0.801, and macro-averaged MCC 0.777, approaching human annotator performance. GPT-5.2 showed the lowest false-negative count per note but more false-positive assignments than either human annotator, reflecting a sensitive but more inclusive annotation profile. Llama-3.1 8B performed competitively among automated methods, whereas HPO-based extraction tools and BioClinical ModernBERT showed lower performance on this low-resource note-level task. Secondary review of GPT-5.2 discordant assignments found no clear hallucinations and suggested that some apparent false positives reflected phenotype evidence missed in the human-derived reference set. **Conclusions**: GPT-5.2 achieved near-human performance for document-level recognition of MS phenotype categories from narrative neurology notes. Direct note-level abstraction may provide a scalable approach for research and population-health phenotyping of large EHR note corpora.

## 1. Introduction

The phenotype of multiple sclerosis (MS) can be described in terms of both disease course and clinical presentation [1,2,3]. Whereas the disease course phenotype distinguishes relapsing-remitting from progressive patterns, the clinical presentation phenotype describes the neurologic signs and symptoms of an individual patient, including motor, sensory, visual, cerebellar, brainstem, sphincter, and cognitive manifestations [3,4,5]. In routine care, the clinical presentation phenotype of MS is typically documented in free-text neurology notes, creating challenges for systematic extraction and comparison across patients [6].

Extraction of the clinical presentation phenotype from electronic health records (EHRs) is important for MS research and routine clinical care [6]. In both clinical trials and patient care, assessment of symptom burden is central to evaluating the response to disease-modifying therapy and informing therapeutic decision-making [7,8]. Swetlik et al. [9] have recommended including standardized discrete data elements in each clinical note (e.g., relapse status, disease course, initial presentation, etc.), but such recommendations have not been widely adopted, and substantial relevant information remains embedded in clinician notes as unstructured free text.

The automated identification and extraction of patient phenotypes from EHRs has emerged as a central problem in biomedical informatics, owing to the large amount of clinically relevant information that resides in unstructured narrative text rather than in standardized discrete fields [10,11]. Such phenotypic data support cohort discovery, longitudinal modeling, and secondary use of clinical data for observational and translational research. In the MS domain, prior work has demonstrated that detailed disease traits, including clinical subtypes and disability measures, can be extracted from routine electronic records with high reliability [6]. However, precise phenotype characterization in most settings still relies heavily on manual review of clinician notes, which is labor-intensive and difficult to scale.

Prior work has shown that high levels of inter-rater agreement can be achieved for neurologic concept annotation in clinical notes when structured annotation tools are used with clear guidelines. Oommen et al. [12] reported that agreement between human annotators for neurological signs and symptoms exceeded agreement between a convolutional neural network and human annotators, underscoring the difficulty of automated neurologic concept annotation.

Traditional approaches to phenotype extraction from clinical text have relied on rule-based systems, dictionary matching, or supervised machine learning [13,14]. More recent approaches have incorporated neural networks and transformer encoders. Although these methods can be effective for well-defined extraction tasks, they may be limited when phenotype recognition requires integration of contextual information distributed across a clinical note. In contrast, large language models (LLMs) can perform zero-shot inference from natural-language task instructions, leveraging extensive pretraining on general and biomedical text to interpret clinical narratives without task-specific fine-tuning [15]. This capability is particularly relevant for MS phenotype identification, where clinical presentation is heterogeneous and often described indirectly rather than through standardized terminology; for example, a note may describe a patient as “clumsy on finger-to-nose testing” rather than explicitly stating “ataxia.” Accordingly, zero-shot LLM-based approaches may provide a scalable alternative for extracting clinically meaningful phenotypes from unstructured narrative documentation.

Because clinical interpretation of narrative documentation involves subjective judgment, human inter-rater reliability provides an important benchmark for evaluating automated annotation systems. Cohen’s κ is widely used to quantify agreement beyond chance and remains a standard measure in clinical annotation studies [16,17]. Establishing the level of agreement achievable between trained human annotators for MS phenotype features is therefore essential for interpreting the performance of automated methods. Perfect concordance is unlikely in either human–human or human–machine comparisons, particularly for phenotypes that are rare, ambiguously documented, or variably expressed in clinical language. Human–human agreement should therefore be interpreted as a practical benchmark rather than as a strict upper bound, because automated systems may exceed individual annotators on some dimensions, particularly recall, while still differing in their false-positive error profiles.

Large language models (LLMs) have demonstrated strong performance across a range of medical natural language processing and clinical reasoning tasks, often without task-specific fine-tuning [18,19]. Related large clinical language models and biomedical transformers have also shown the value of domain-specific pretraining for medical NLP [20]. More recent work applying LLMs to high-throughput phenotyping of physician notes suggests that LLM-based approaches can outperform traditional deep learning and classical machine learning methods for some clinical phenotyping tasks [21]. These models may enable scalable extraction of phenotypes and disease status directly from narrative EHR documentation in zero-shot or low-supervision settings.

In this study, phenotype identification was performed at the level of the complete clinical note rather than at the level of individual text spans. The task was document-level phenotype recognition: for each note, the annotator or model determined whether each of 17 clinically meaningful phenotype categories was present or absent anywhere in the document. The task did not require localization of the exact supporting text span, counting repeated mentions, normalization to a canonical ontology term, or assignment of a machine-readable ontology identifier.

This distinction is important because many biomedical NLP pipelines combine several separable tasks under the broad label of concept or phenotype extraction. Span-level named entity recognition identifies the location of a relevant mention in text. Concept normalization maps that refer to a canonical term in a controlled vocabulary such as SNOMED CT or the Human Phenotype Ontology (HPO). Identifier linking assigns the corresponding ontology code. Each of these steps introduces distinct sources of variability and error. Prior work has shown that LLMs may accurately interpret biomedical or clinical concepts while failing to reliably retrieve the corresponding standardized identifiers [22,23,24]. By restricting the primary task to high-level note-level phenotype classification, the present study isolates clinical interpretation from the downstream challenge of ontology normalization.

The central question addressed in this study is whether a large language model can read a complete neurology note and arrive at the same high-level clinical interpretation of MS phenotype features as a human observer, as measured by inter-rater agreement and performance relative to an adjudicated reference annotation set. We additionally compare a frontier LLM (GPT-5.2) with a locally run open-source instruction-tuned LLM (Llama-3.1 8B), a supervised transformer encoder (BioClinical ModernBERT), and several HPO-based phenotype extraction tools (Doc2Hpo, PhenoSnap, and ClinPhen). Because the HPO-based tools produce more granular span- or concept-level ontology outputs, their results were post-processed into the same 17-category note-level representation used for human and LLM annotation.

## 2. Methods

### 2.1. Neurology Notes

A total of 12,661 de-identified neurology clinical notes from the University of Illinois Hospital, the primary teaching hospital of UI Health, were obtained from a REDCap database for the period 14 January 2016 through 2 September 2022. Notes were filtered to include only those longer than 600 words, associated with a diagnosis of multiple sclerosis (ICD-10-CM G35), and classified as Progress Note encounters. After deduplication, 4617 notes remained for analysis.

Each clinical note represents a narrative summary of a patient encounter and serves as the unit of analysis in this study. Prior to annotation, notes were converted to JavaScript Object Notation (JSON) format, with each complete note represented as a single JSON object. This note-level representation preserves the full clinical context of the note, enabling evaluation of disease status and phenotype-feature identification as a task of clinical interpretation rather than isolated text-span extraction. Use of the de-identified clinical documentation for research was approved by the Institutional Review Board of the University of Illinois (Protocol No. 2017-0520Z).

### 2.2. Human Annotation Process

Human annotation was performed using Prodigy (version 1.18.0., Explosion AI, Berlin, Germany), an annotation platform for natural language processing workflows. Prodigy provides a locally hosted web interface and integrates with the spaCy library. Each clinical note was presented as a single annotation unit. This study was designed as an inter-rater agreement experiment on a subset of 100 notes, randomly sampled from 4617 eligible MS progress notes. The objective was to estimate human–human and human–LLM agreement under controlled conditions rather than to perform large-scale annotation.

Two annotators independently labeled the notes: Annotator 1 (A1), a pre-medical student, and Annotator 2 (A2), a senior neurologist, enabling assessment across differing levels of clinical expertise.

Written definitions of phenotype features (Appendix C) were provided before annotation, and joint training sessions using representative notes were conducted to promote consistency. Disease diagnoses (e.g., multiple sclerosis, optic neuritis) were not annotated, and modifiers such as laterality, severity, and duration were not separately coded.

Annotation was performed using Prodigy’s textcat.manual recipe. Seventeen phenotype features (Appendix A) were annotated using a multi-label text classification interface, allowing multiple non-exclusive categories to be assigned to each complete note. In this annotation mode, Prodigy records document-level category assignments rather than start and end character offsets for individual text spans. Annotations were stored in an SQLite database and exported in JSON format for analysis.

The annotation framework, therefore, operated at the level of the complete clinical note. Phenotypes were recorded as present or absent, without counting repeated mentions, localizing exact text spans, or normalizing mentions to ontology identifiers. Span-based named entity recognition was not performed.

### 2.3. Annotation by LLM

The same set of 100 clinical notes used for human annotation was independently evaluated using a frontier large language model, GPT-5.2 (OpenAI; gpt-5.2-2025-12-11), accessed via the OpenAI Responses API. Hereafter, we refer to this model as “the LLM.” The temperature was set to 0 to minimize sampling variability, and no explicit maximum token limit was specified; default model settings were used for response length. The task was framed as multi-label classification at the level of the complete clinical note, using the same phenotype definitions provided to human annotators. Each note was processed independently using a fixed prompt. Model outputs were constrained using a predefined JSON schema to ensure structured and reproducible annotations. For each note, the model returned a list of phenotype items, each containing

- label: phenotype category (from predefined list)
- present: boolean indicating presence or absence
- evidence: supporting text excerpt from the note
- detail: optional clarification

An optional warning field was included to capture uncertainty or ambiguity in the model output. The full prompt, label definitions, and implementation details are provided in Appendix A, Appendix B and Appendix C.

To assess the robustness of LLM annotation to prompt formulation, we performed a prompt-sensitivity analysis. We repeated annotation under alternative prompt conditions that varied three components of the LLM configuration: (1) temperature setting, (2) length and specificity of phenotype definitions, and (3) length and specificity of annotation rules. These experiments were intended to evaluate whether performance was strongly dependent on a particular prompt wording rather than to optimize the prompt. Each sensitivity run used the same predefined 10-note subset, the same 17 phenotype categories, and the same structured JSON output schema. Performance was compared using the same macro- and micro-averaged precision, recall, F1, and Matthews correlation coefficient metrics used in the primary analysis.

### 2.4. Adjudication Process to Create the Reference Annotation Set

A reference annotation set was constructed through a structured adjudication process to resolve discrepancies between annotators. The dataset comprised 100 clinical notes and 17 phenotypic features, yielding 1700 note–feature evaluations. For 1535 note–feature pairs (90.3%), both human annotators (A1 and A2) were in agreement, and the reference label was assigned directly based on this consensus. In 165 cases (9.7%), the human annotators were discordant. These discordant cases were re-reviewed and adjudicated by the senior neurologist (A2). Adjudication resulted in agreement with A2’s original label in 93 cases and with A1’s original label in 72 cases. This process yielded a complete adjudicated reference annotation set comprising 1700 note–feature labels.

### 2.5. Review of GPT-5.2 Assignments Discordant with Reference Assignments

After the primary adjudicated reference annotation set was finalized, we performed a post hoc secondary review of GPT-5.2 assignments that were discordant with the reference set. This review was conducted to characterize apparent GPT-5.2 false positives and to distinguish hallucination from other sources of disagreement, including unsupported inference and potential omissions in the human-derived reference set. The review was not used to modify the primary reference set or to recompute the primary performance metrics.

For each apparent false-positive GPT-5.2 note–category assignment, we reviewed the model-provided evidence field and the corresponding clinical note context. Apparent false positives were categorized qualitatively as negation or symptom-resolution errors, historical-current conflation, medication-based inference, weakly supported inference from indirect evidence, or likely valid phenotype evidence not incorporated into the reference set. Apparent false-negative assignments were not reviewed in this secondary analysis because the absence of GPT-5.2 labels did not include model-provided evidence fields. This secondary review was used only for error characterization and interpretation of the primary results.

### 2.6. Comparator Methods

The adjudicated reference annotation set was used as the ground truth for evaluating automated comparator methods on the 17-category note-level phenotype classification task. Comparator methods were selected to represent three approaches to clinical phenotype extraction: HPO-based concept extraction tools, instruction-following LLMs, and supervised transformer encoders. The HPO-based tools included Doc2Hpo [25], ClinPhen [6], and PhenoSnap [26,27]. The open-source LLM comparator was Llama-3.1 8B Instruct, run locally through Ollama. The supervised transformer comparator was BioClinical ModernBERT [28].

Doc2Hpo was evaluated as an HPO-based concept-extraction comparator. We used the standard Doc2Hpo API endpoint (https://doc2hpo.wglab.org/ (Accessed on 22 May 2026)) to process the clinical notes. Doc2Hpo accepts free-text input and returns recognized Human Phenotype Ontology (HPO) terms, ontology identifiers, and span-level information, including detected text position and span length [25]. The tool was run with default settings, including matching against HPO terms and synonyms within the Phenotypic Abnormality subtree (HP:0000118), negation detection, and longest-match selection for overlapping annotations. Doc2Hpo therefore represents a traditional HPO-oriented extraction system that identifies span-level phenotype concepts and maps them to ontology terms and identifiers.

ClinPhen was evaluated as a rule-based HPO phenotype extraction comparator. We downloaded and installed ClinPhen locally and ran it on macOS. Each of the 100 MS progress notes was passed to ClinPhen as a separate free-text file. ClinPhen extracts phenotypes from clinical notes by mapping text to HPO terms. Its pipeline segments notes into sentences and subsentences, lemmatizes words, matches phenotype names and synonyms, and applies rule-based filters to exclude likely false-positive mentions, including negated findings and mentions referring to family members rather than the patient. ClinPhen returns HPO phenotype terms sorted by frequency of occurrence, first mention position, and HPO identifier.

PhenoSnap was evaluated as an additional HPO-based phenotype extraction comparator. Because no peer-reviewed manuscript describing PhenoSnap and no formal software release number were available at the time of analysis, we used the open-source implementation available from the WGLab GitHub repository at https://github.com/WGLab/PhenoSnap (Accessed on 22 May 2026; commit a4199d55147347ffc9a26b9e3d282448b9b04774). The repository was downloaded and run locally on macOS. PhenoSnap performs local phenotype extraction from free text using spaCy and a locally downloaded HPO OBO file, without cloud-based LLMs or remote APIs. The tool uses phrase matching against HPO labels and synonyms, includes dependency-based negation detection, and outputs matched phenotype mentions with HPO identifiers, labels, character offsets, and negation status.

For all three HPO-based systems, native span- or concept-level outputs were post-processed into the same 17-category note-level representation used for human and LLM annotation. HPO terms and identifiers returned by these systems were mapped to the corresponding high-level phenotype categories using the curated lookup table described below. A phenotype category was coded as present for a note if one or more mapped HPO terms assigned to that category were detected.

To evaluate whether a smaller open-source LLM could perform the same note-level phenotype abstraction task, we ran Llama-3.1 8B Instruct locally using Ollama version 0.24.0 on macOS. The model was accessed via the local Ollama chat API using the model tag llama3.1:8b (model ID 46e0c10c039e; 8.0B parameters; Q4_K_M quantization), with a temperature of 0.0. The same 100 MS notes, phenotype definitions, and structured JSON output schema used for GPT-5.2 were used without fine-tuning, prompt optimization, or retrieval-augmented generation. Each note was processed independently as a complete note-level multilabel classification task. Model outputs were normalized to the same 17 binary phenotype categories and converted to a note-feature matrix for comparison with the adjudicated reference annotation set.

To include a contemporary supervised transformer baseline, we evaluated BioClinical ModernBERT (https://huggingface.co/collections/thomas-sounack/bioclinical-modernbert, Acessed on 22 May 2026) using the Hugging Face Transformers framework. Unlike instruction-following LLMs, BioClinical ModernBERT is a pretrained clinical transformer encoder and does not directly perform phenotype classification without task-specific supervision. We therefore added a randomly initialized multilabel sequence-classification head with 17 output nodes, corresponding to the 17 phenotype categories, and fine-tuned the model on the annotated notes.

Evaluation was performed using 5-fold cross-validation with random shuffling and a fixed random seed. In each fold, the model was trained on 80 notes and evaluated on 20 held-out notes. The maximum input length was set to 4096 tokens to reduce truncation of long clinical notes; the model configuration supported sequences up to 8192 tokens. Training used 3 epochs, learning rate $2\times 10^{-5}$, weight decay 0.01, and batch size 1. Multilabel predictions were generated by applying a sigmoid transformation to the model logits and thresholding probabilities at 0.30. Macro- and micro-averaged precision, recall, F1, and Matthews correlation coefficient were computed across folds.

### 2.7. Mapping to High-Level Categories

Human annotators, GPT-5.2, Llama-3.1 8B, and BioClinical ModernBERT generated outputs directly as the 17 predefined note-level phenotype categories. In contrast, ClinPhen, Doc2Hpo, and PhenoSnap generated granular concept-level outputs as Human Phenotype Ontology (HPO) terms and identifiers. To enable comparison across methods, outputs from these HPO-based systems were mapped to the same 17 high-level phenotype categories used for human and LLM annotation.

Across the HPO-based systems, 382 unique HPO term–identifier pairs were identified. Each unique pair was manually reviewed using a curated lookup table. Of these, 147 were mapped to one of the 17 predefined phenotype categories, whereas 235 did not correspond to any target category and were excluded from the 17-category note-level grid.

For each note, a phenotype category was coded as present if one or more HPO terms mapped to that category were detected by the extraction system; otherwise, the category was coded as absent. False positives and false negatives were defined at the note–category level. A false positive occurred when a comparator method assigned a phenotype category as present for a note but that category was absent in the adjudicated reference annotation set. A false negative occurred when a phenotype category was present in the reference set but was not assigned by the comparator method. HPO terms that did not map to any of the 17 target categories were outside the predefined scoring schema and were therefore not counted as false positives.

### 2.8. Statistical Analysis

Agreement was assessed at the note–category level, with each clinical note contributing 17 binary phenotype decisions. Human–human and human–LLM agreement were summarized using unadjusted percent agreement and Cohen’s κ [16,17]. Each annotation method, including A1, A2, GPT-5.2, Llama-3.1 8B, BioClinical ModernBERT, Doc2Hpo, ClinPhen, and PhenoSnap, was compared with the adjudicated reference annotation set.

For each phenotype category, true positives, false positives, false negatives, and true negatives were computed relative to the reference set. Precision, recall, F1 score, and Matthews correlation coefficient (MCC) were then calculated using standard definitions. Macro-averaged metrics were computed as the unweighted mean across the 17 phenotype categories. Micro-averaged metrics were computed by pooling all note–category decisions across phenotypes. False-positive and false-negative rates were additionally summarized as mean false-positive and false-negative phenotype assignments per note.

All analyses were performed in Python (3.10.20) using pandas (2.3.3), scikit-learn (1.7.1), matplotlib (3.10.9), seaborn (0.13.2), json, collections, requests, and the openai (2.30.0) packages.

Global performance metrics for the human annotators and automated comparator methods were computed against the adjudicated reference annotation set. Macro-averaged metrics are unweighted means across the 17 phenotype categories. Micro-averaged metrics are pooled across all note–category decisions. MCC denotes the Matthews correlation coefficient computed over pooled note–category decisions. Values in parentheses are note-level bootstrap 95% confidence intervals. Bootstrap 95% confidence intervals were estimated by resampling notes with replacement. Each bootstrap sample contained the same number of notes as the original analysis for that method, preserving the 17 phenotype decisions within each sampled note. Metrics were recomputed for 5000 bootstrap samples, and the 2.5th and 97.5th percentiles were used as confidence limits. MCC was computed over all pooled note–category decisions (Appendix D, Table A1, Table A2 and Table A3).

## 3. Results

### 3.1. Concordance Between Human Annotators and the LLM

We first examined pairwise agreement among the two human annotators and GPT-5.2 on the 100-note annotation set. Each note was annotated for the presence or absence of 17 predefined neurological phenotype features, including weakness, sensory symptoms, cognitive symptoms, gait impairment, pain, and falls. Agreement was assessed at the note–phenotype level using both raw percent agreement and Cohen’s κ.

Human–human agreement was generally high by raw agreement, with percent agreement ranging from 0.79 to 0.98 across phenotype categories (Table 1). Cohen’s κ was moderate to substantial for most categories, but lower for several infrequent or more difficult-to-define features, including dysphagia, ataxia, bowel symptoms, spasticity, and hyperreflexia. The low or negative κ values for rare categories should be interpreted cautiously because κ is sensitive to class prevalence.

GPT-5.2 was then evaluated in a zero-shot prompting mode using the same note-level phenotype definitions and without fine-tuning or retrieval-augmented generation. Pairwise agreement between GPT-5.2 and each human annotator was broadly comparable to human–human agreement (Table 2 and Table 3). As with human–human agreement, concordance varied by phenotype category, with lower agreement for categories that were infrequent, clinically ambiguous, or inconsistently documented.

### 3.2. Performance of Annotators and Comparators Against the Adjudicated Reference Set

We next evaluated each human annotator and automated comparator against the adjudicated reference annotation set. GPT-5.2 achieved the strongest automated performance, with macro-F1 of 0.801 and macro-averaged MCC of 0.777, closely approximating Annotator 1 and approaching the performance of Annotator 2 (Table 4). GPT-5.2 showed the highest macro-recall of all methods, consistent with a sensitive annotation profile. Annotator 2 showed the highest macro-precision and macro-F1, consistent with a more conservative labeling pattern. Llama-3.1 8B also outperformed the HPO-based extraction tools and BioClinical ModernBERT, although with lower precision than GPT-5.2. The HPO-based tools showed lower overall performance than the LLMs and human annotators. BioClinical ModernBERT showed limited performance in this low-resource supervised setting, likely reflecting limited task-specific training data. Macro and micro-averaged metrics with 95% CIs are available in the Appendix D (Table A1, Table A2 and Table A3).

**Figure 1:**
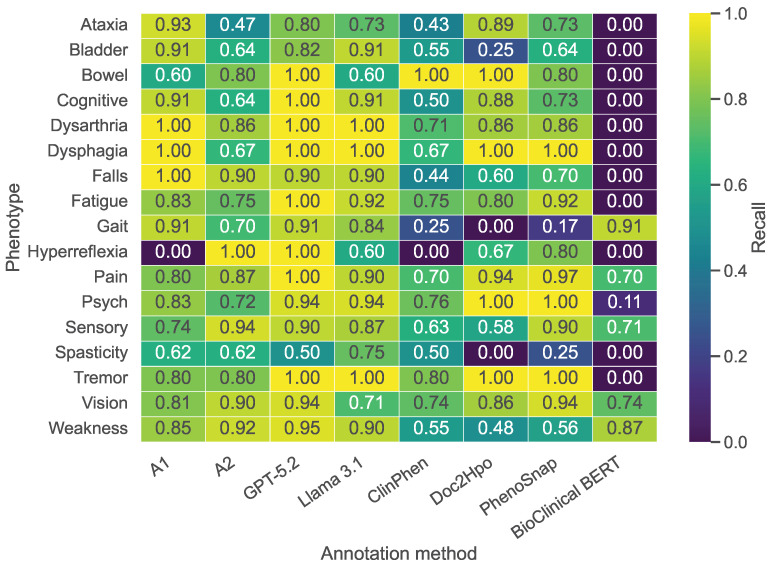
Phenotype-level recall across annotation methods relative to the adjudicated reference annotation set. Recall represents the proportion of reference-positive note–phenotype assignments detected by each method. Lower recall indicates a greater tendency toward false-negative phenotype omissions.

The recall heat map demonstrated that method performance varied substantially across phenotype categories (Figure 1). GPT-5.2 maintained high recall across both discrete findings and broader functional categories, whereas Llama-3.1 8B showed a similar recall-oriented pattern. The HPO-based extraction tools performed best for lexically specific phenotypes such as dysarthria, dysphagia, tremor, and vision abnormalities, but had more difficulty with broader or more variably expressed categories such as weakness, gait impairment, bladder symptoms, falls, and cognitive symptoms. These results suggest that span- and ontology-oriented tools may be less effective when phenotype recognition requires note-level semantic abstraction rather than detection of a specific term or synonym.

**Figure 2:**
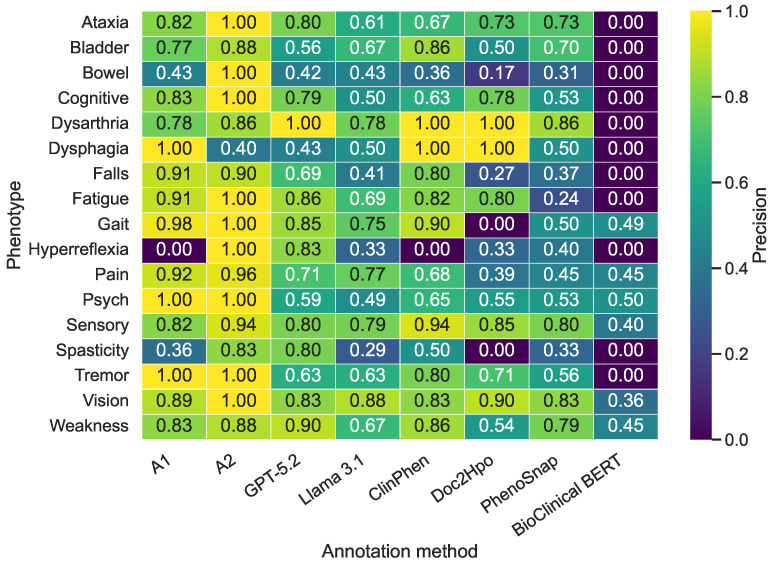
Phenotype-level precision across annotation methods relative to the adjudicated reference annotation set. Precision represents the proportion of predicted note–phenotype assignments that were present in the reference set. Lower precision indicates a greater tendency toward false-positive phenotype assignments.

The precision heat map demonstrated distinct false-positive profiles across methods (Figure 2). Annotator 2 had the most consistently high precision, whereas GPT-5.2 maintained high precision for many phenotype categories while also showing evidence of overinclusive assignment in some broader or more inferential categories. Llama-3.1 8B showed lower and more variable precision than GPT-5.2. The HPO-based extraction tools had heterogeneous precision across categories, reflecting the difficulty of mapping granular HPO term outputs to broad note-level phenotype categories. BioClinical ModernBERT showed low precision across many categories, consistent with limited learning from the small supervised training set.

### 3.3. False Positives and False Negatives by Annotation Method

**Figure 3:**
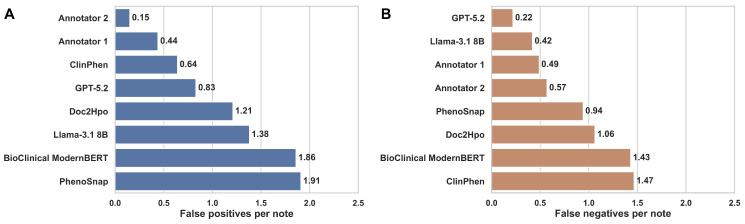
False-positive and false-negative phenotype assignments per note by annotation method. Panel (**A**) shows the mean number of false-positive phenotype assignments per note (blue bars). Panel (**B**) shows the mean number of false-negative phenotype omissions per note (orange bars). Each note included 17 binary phenotype decisions.

False-positive and false-negative error counts per note provided a clinically interpretable view of method-specific error profiles (Figure 3). Annotator 2 showed the most conservative pattern, with the fewest false-positive phenotype assignments per note but more false-negative omissions than GPT-5.2. In contrast, GPT-5.2 had the lowest false-negative count per note of all methods, consistent with its high recall, but generated more false-positive assignments than either human annotator. Llama-3.1 8B showed a similar recall-oriented pattern but with a higher false-positive burden. These findings indicate that the main difference between human and LLM annotation was not simply overall accuracy, but the balance between omission errors and overinclusive phenotype assignment.

### 3.4. Prompt Sensitivity Analysis

Prompt-sensitivity experiments were performed on a predefined 10-note subset and were intended as a robustness check rather than as prompt optimization. Across variations in temperature, rule length, and definition length, performance differences were modest and did not suggest strong dependence on a single prompt formulation (Table 5).

### 3.5. Review of GPT-5.2 Discordant Assignments

A secondary review was performed for GPT-5.2 assignments that were discordant with the adjudicated reference annotation set (Table 6). GPT-5.2 had 22 false-negative and 83 apparent false-positive note–category assignments in the primary analysis. Review of the apparent false positives did not identify clear hallucinations. Instead, discordances reflected negation errors, medication-based inference, weak inference from indirect evidence, historical-current conflation, and cases in which the GPT-5.2 assignment appeared likely valid despite being absent from the reference set. The largest subgroup consisted of likely valid assignments (45 of 83 apparent false positives), suggesting that the measured GPT-5.2 precision may be conservative relative to the human-derived reference standard.

## 4. Discussion

This study evaluated whether large language models can perform note-level recognition of clinically meaningful MS phenotype categories from narrative neurology notes. The principal finding is that GPT-5.2 achieved the strongest automated performance and approached the performance of human annotators when evaluated against an adjudicated reference annotation set. GPT-5.2 showed particularly high recall, identifying most reference-positive phenotype categories across the 100-note set. Its macro-F1 and macro-averaged MCC were close to those of Annotator 1 and approached those of Annotator 2, the senior neurologist. These results suggest that frontier LLMs can perform clinically meaningful document-level phenotype recognition in a zero-shot setting.

The performance pattern differed between human and LLM annotations. Annotator 2 showed the most conservative profile, with the highest precision and the fewest false-positive assignments per note, but more false-negative omissions than GPT-5.2. In contrast, GPT-5.2 showed the lowest false-negative count per note and the highest recall, but generated more apparent false positives than either human annotator. This tradeoff is clinically plausible. Manual annotation of complete clinical notes across 17 simultaneous phenotype categories imposes a substantial vigilance burden on human annotators, creating opportunities for omission errors. LLMs are not subject to vigilance decrement in the same way and may therefore apply the annotation schema more exhaustively. However, this advantage was offset by a greater tendency toward overinclusive assignment.

The qualitative review of GPT-5.2 discordant assignments provided further insight into this error profile. We did not identify clear hallucinations among the apparent false-positive assignments. Instead, discordances reflected recognizable mechanisms, including negation or symptom-resolution errors, medication-based inference, weak inference from indirect evidence, historical–current state conflation, and cases in which GPT-5.2 appeared to identify valid phenotype evidence that had not been incorporated into the human-derived reference set. The largest subgroup consisted of likely valid assignments that had been missed during human annotation or adjudication. This finding suggests that the measured precision of GPT-5.2 may be conservative, because some apparent false positives may reflect omissions in the reference annotation set rather than true model errors. Importantly, we did not recompute the primary performance metrics after this secondary review, and the original adjudicated reference set remained the basis for all primary analyses.

The comparator analyses help place the GPT-5.2 results in context. Llama-3.1 8B, run locally through Ollama without fine-tuning or retrieval-augmented generation, performed substantially better than the HPO-based extraction systems and the supervised transformer comparator, although below GPT-5.2. This finding suggests that note-level phenotype abstraction is not unique to proprietary frontier models, but that model scale and capability remain important. The performance of Llama-3.1 8B is notable because it was run locally using the same structured prompt and output schema as GPT-5.2. Locally executable LLMs may therefore be attractive in settings where cost, privacy, reproducibility, or infrastructure constraints limit reliance on cloud-based APIs.

The HPO-based tools, including Doc2Hpo, ClinPhen, and PhenoSnap, performed less well on the present task. This should not be interpreted simply as a failure of those systems. They were designed primarily for span- or concept-level phenotype extraction and ontology linking, not for broad note-level classification into our 17 MS phenotype categories. Their native outputs include HPO terms and identifiers, which are more granular than the target labels used in this study. To compare them with human and LLM annotations, we mapped their HPO outputs to the 17 high-level categories. This post-processing necessarily changed the nature of their native task. Nonetheless, the results highlight an important practical limitation: tools optimized for lexical or ontology-based span extraction may miss phenotype evidence when it is expressed indirectly, distributed across the note, or described in clinical-functional terms rather than as a canonical ontology label. For example, a note may support gait impairment through descriptions of cane use, poor balance, or difficulty ambulating, even if it does not contain an exact phrase that maps cleanly to a specific HPO gait term.

BioClinical ModernBERT showed limited performance, likely reflecting the small number of notes available for supervised training. This result should be interpreted cautiously. BioClinical ModernBERT is a pretrained transformer encoder, not an instruction-following generative model. To apply it to the present task, we added a randomly initialized 17-label classification head and fine-tuned the model on the annotated notes. In 5-fold cross-validation, each fold provided only 80 training notes, with sparse positive examples for several phenotype categories. Thus, the limited performance likely reflects the difficulty of fine-tuning a supervised classifier in a low-resource setting (limited training examples) rather than a lack of clinically useful representations in the pretrained encoder. By contrast, instruction-following LLMs (GPT 5.2 and Llama 3.1 8B) can apply natural-language task definitions directly, which may make them more practical when limited training examples are available.

These findings support a distinction between document-level phenotype recognition and conventional span-level concept extraction. Many biomedical NLP pipelines, including tools such as ClinPhen, Doc2Hpo, and PhenoSnap, decompose phenotyping into sequential steps: span detection, concept normalization, ontology identifier assignment, and aggregation into analysis-ready variables. This fine-grained approach is essential when exact mention localization, ontology alignment, or knowledge representation is the primary goal. The present study addressed a different task: determining whether a clinically meaningful phenotype category was present in any part of the complete note. For this document-level task, exact span boundaries and ontology identifiers were not required. Instead, the relevant challenge was clinical interpretation: whether the note supported a high-level phenotype such as gait impairment, weakness, pain, cognitive symptoms, bladder dysfunction, or hyperreflexia. Thus, the unit of analysis was the clinical note, not the individual phenotype mentioned.

The note-level approach may be particularly useful for population-health and research applications. Large EHR corpora contain thousands or millions of narrative notes that cannot feasibly be annotated manually. Converting these notes into lower-dimensional phenotype variables could support cohort discovery, epidemiologic studies, longitudinal outcome tracking, real-world evidence generation, quality improvement initiatives, and downstream machine learning. In this setting, the goal is not to replace clinical judgment for an individual patient, but to create scalable, interpretable variables for aggregate analysis. This intended use is distinct from real-time clinical decision support or individualized precision medicine, where false positives and false negatives may have immediate consequences and would require prospective validation, monitoring, and human oversight.

The prompt-sensitivity analysis suggested that GPT-5.2’s performance was not highly dependent on a narrowly optimized prompt formulation. Across variations in temperature, rule length, and phenotype-definition length on a predefined 10-note subset, performance differences were modest. This finding should be interpreted cautiously because the sensitivity analysis was small, but it suggests that the main result was not simply an artifact of a highly tuned prompt. Future work should examine whether additional rules, such as explicitly prohibiting phenotype inference from medication use alone, can reduce false positives without compromising recall. This study has several limitations. First, the dataset was small: only 100 notes were manually annotated, although each note contributed 17 binary phenotype decisions. No formal power calculation was performed. The sample was sufficient for a controlled inter-rater agreement and proof-of-concept comparator study, and uncertainty was estimated using note-level bootstrap confidence intervals. However, larger datasets are needed to obtain more stable phenotype-specific estimates, particularly for rare categories such as hyperreflexia, spasticity, dysphagia, and bowel symptoms.

Second, the reference annotation set was derived from two human annotators, and discordant labels were adjudicated by A2, who was also one of the original annotators. An independent adjudication committee would have been preferable and may have reduced potential incorporation bias. Although 90.3% of note–feature pairs were concordant before adjudication, this limitation should be considered when interpreting human–machine comparisons.

Third, the study was performed on MS progress notes from a single academic health system. Notes shorter than 600 words were excluded because they were often task-specific encounters that lacked a complete history and neurological examination. Performance may differ in other institutions, specialties, note types, EHR templates, shorter notes, or patient populations. Fourth, the HPO-based comparators did not produce usable outputs for all notes in our workflow, and the causes of failure were not systematically investigated. Fifth, the supervised transformer comparator was limited by the small number of training examples. The BioClinical ModernBERT results should therefore not be interpreted as a definitive benchmark of supervised transformer performance, but as an illustration of the challenge of training such models under low-resource conditions. Sixth, the secondary review of GPT-5.2 discordant assignments was exploratory and was not used to revise the primary reference set or recompute the main performance metrics.

Finally, this study did not evaluate the full ontology normalization pipeline. We did not require exact span localization, canonical HPO term selection, SNOMED CT mapping, or ontology identifier assignment. Strong performance on note-level phenotype recognition should therefore not be interpreted as evidence that LLMs can reliably perform fine-grained ontology normalization or identifier linking. Conversely, the lower performance of HPO-based systems in this study should not be interpreted as evidence that those tools are ineffective for their native span-level or ontology-linking tasks. The methods were evaluated on a high-level note-level phenotype classification endpoint.

In summary, GPT-5.2 performed at a level comparable to human annotators for document-level MS phenotype recognition and achieved particularly high recall across phenotype categories. Llama-3.1 8B demonstrated that smaller locally run instruction-tuned LLMs can also perform this task with useful accuracy, although below GPT-5.2. HPO-based extraction tools and a supervised transformer encoder were less effective for this low-resource note-level classification task. These results support the feasibility of LLM-assisted document-level phenotyping as a potentially scalable approach for research and population-health analysis of large EHR note corpora, while emphasizing the need for careful error characterization, transparent reference standards, and prospective validation before use in clinical decision support.

## 5. Conclusions

GPT-5.2 achieved near-human performance for document-level recognition of MS phenotype categories from narrative neurology notes, with particularly high recall. A locally run open-source LLM, Llama-3.1 8B, also performed competitively, whereas HPO-based extraction tools and a supervised transformer encoder were less effective for this low-resource note-level task. These findings support direct note-level abstraction as a feasible approach for scalable research and population-health phenotyping of large EHR note collections. The approach should be viewed as complementary to, rather than a replacement for, span-level ontology extraction and requires further validation before use in clinical decision support. By converting narrative notes into structured note-level phenotype variables, this approach may also enable downstream unsupervised analyses, including dimensionality reduction and phenotype module discovery at scale.

## Figures and Tables

**Table 1:** Pairwise concordance for note-level phenotype categories: A1 vs. A2.

Phenotype	Agreement	κ	A1 Positive	A2 Positive
Falls	0.97	0.84	11	10
Tremor	0.98	0.74	4	4
Dysarthria	0.96	0.73	9	7
Vision	0.88	0.70	28	28
Psych	0.92	0.67	15	13
Fatigue	0.94	0.67	11	9
Pain	0.87	0.67	26	27
Gait	0.82	0.61	40	30
Cognitive	0.93	0.60	12	7
Sensory	0.83	0.59	28	31
Weakness	0.79	0.56	40	41
Bladder	0.91	0.52	13	8
Dysphagia	0.96	0.48	3	5
Ataxia	0.88	0.45	17	7
Bowel	0.93	0.33	7	4
Spasticity	0.84	0.13	14	6
Hyperreflexia	0.94	−0.02	1	5

Agreement denotes raw percent agreement. Cohen’s κ adjusts for chance agreement but is sensitive to the prevalence of phenotypes; low or negative values may occur for rare categories despite high raw agreement.

**Table 2:** Pairwise concordance for note-level phenotype categories: A1 vs. GPT-5.2.

Phenotype	Agreement	κ	A1 Positive	LLM Positive
Dysarthria	0.98	0.86	9	7
Fatigue	0.97	0.86	11	14
Cognitive	0.96	0.82	12	14
Falls	0.94	0.72	11	13
Ataxia	0.92	0.70	17	15
Weakness	0.85	0.69	40	41
Gait	0.84	0.68	40	46
Vision	0.85	0.65	28	35
Tremor	0.96	0.65	4	8
Pain	0.82	0.61	26	42
Bowel	0.93	0.60	7	12
Dysphagia	0.96	0.58	3	7
Bladder	0.89	0.56	13	16
Psych	0.84	0.55	15	29
Sensory	0.79	0.52	28	35
Spasticity	0.83	0.03	14	5
Hyperreflexia	0.93	−0.02	1	6

Agreement denotes raw percent agreement. Cohen’s κ adjusts for chance agreement but is sensitive to the prevalence of phenotypes; low or negative values may occur for rare categories despite high raw agreement.

**Table 3:** Pairwise concordance for note-level phenotype categories: A2 vs. GPT-5.2.

Phenotype	Agreement	κ	A2 Positive	LLM Positive
Hyperreflexia	0.99	0.90	5	6
Dysarthria	0.98	0.85	7	7
Sensory	0.90	0.77	31	35
Fatigue	0.95	0.76	9	14
Weakness	0.88	0.75	41	41
Vision	0.89	0.75	28	35
Falls	0.93	0.66	10	13
Tremor	0.96	0.65	4	8
Pain	0.83	0.63	27	42
Cognitive	0.93	0.63	7	14
Gait	0.82	0.63	30	46
Bladder	0.92	0.63	8	16
Psych	0.84	0.54	13	29
Spasticity	0.95	0.52	6	5
Ataxia	0.90	0.50	7	15
Dysphagia	0.94	0.47	5	7
Bowel	0.92	0.47	4	12

Agreement denotes raw percent agreement. Cohen’s κ adjusts for chance agreement but is sensitive to phenotype prevalence; low or negative values may occur for rare categories despite high raw agreement.

**Table 4:** Annotator metrics for concept recognition task (macro averages).

Method	Precision	Recall	F1	MCC
BioClinical ModernBERT	0.156	0.238	0.174	0.071
Doc2Hpo	0.560	0.694	0.586	0.534
PhenoSnap	0.555	0.762	0.608	0.537
ClinPhen	0.723	0.587	0.621	0.567
Llama-3.1 8B	0.599	0.852	0.690	0.643
Annotator 1	0.779	0.797	0.782	0.750
GPT-5.2	0.734	0.921	0.801	0.777
Annotator 2	0.920	0.776	0.830	0.815

**Table 5:** Prompt-sensitivity analysis on a predefined 10-note subset. Values represent macro-averaged precision, recall, and F1 relative to the adjudicated reference annotation set.

Option	Precision	Recall	F1
Temperature 0.0	0.612	0.583	0.590
Temperature 0.5	0.631	0.672	0.638
Temperature 1.0	0.612	0.613	0.609
Rules (Long)	0.612	0.583	0.590
Rules (Short)	0.612	0.613	0.609
Definitions (Long)	0.612	0.603	0.601
Definitions (Short)	0.617	0.671	0.633

**Table 6:** Review of GPT-5.2 assignments discordant with the adjudicated reference annotation set.

Type	Subtype	Count	Example (Category)
False negative	Not reviewed	22	–
False positive	Total	83	–
	Negation	16	“No ataxia” (Ataxia)
	Medication inference	4	“Baclofen prescribed” (Spasticity)
	Weak inference	12	“On waiting list for therapist” (Psych)
	Historical inference	6	“Depression in past” (Psych)
	Likely valid	45	“Spastic paraparesis” (Weakness, Spasticity)

## Data Availability

The data underlying this study are not publicly available because they consist of de-identified electronic health record clinical notes that remain subject to institutional and regulatory restrictions on sharing. Data may be made available from the corresponding author on reasonable request and with permission of the University of Illinois, where applicable. The Python code used for data processing and analysis is available from the corresponding author upon reasonable request.

## Appendix A. Labels

PHENOTYPE_LABELS = [

  “Ataxia”,

  “Bladder”,

  “Bowel”,

  “Cognitive”,

  “Dysarthria”,

  “Dysphagia”,

  “Falls”,

  “Fatigue”,

  “Gait”,

  “Hyperreflexia”,

  “Pain”,

  “Psych”,

  “Sensory”,

  “Spasticity”,

  “Tremor”,

  “Vision”,

  “Weakness”]

## Appendix B. Prompt

You are labeling a neurology MS note segment for the  PRESENCE/ABSENCE of multiple items.

This is MULTI-LABEL (NOT exclusive):  Multiple labels may be present.

LABEL DEFINITIONS: {LABEL_DEFS}

RULES:

- Use ONLY what is explicitly stated in the TEXT.

- Mark present = true ONLY if there is clear evidence the item applies.

- If present = true: evidence MUST be a short direct quote (<=25 words).

- If present = false: evidence MUST be “” (empty string). detail may be “”.

- Do NOT guess. Do NOT use outside knowledge.

- Output MUST include each label at most once.

Return JSON only, matching the schema exactly.

## Appendix C. Definitions

LABEL_DEFS =  {

  “Psych”: “Psychiatric symptoms (e.g., depression, anxiety, panic, mood disorder)”,

  “Dysarthria”: “Dysarthria, slurred speech, scanning speech.”,

  “Dysphagia”: “Dysphagia, difficulty swallowing, aspiration, feeding tube.”,

  “Spasticity”: (

    “Spasticity or increased tone/hypertonia/spastic gait. ”

    “Do NOT mark for vague stiffness alone.”),

  “Hyperreflexia”: (

    “Hyperreflexia/brisk reflexes/increased DTRs (e.g., 3+ or 4+), ”

    “and/or clonus explicitly mentioned.”),

  “Vision”: (

    “Visual or eye-movement abnormality (blurred vision, visual field defect, ”

    “diplopia, nystagmus, EOM limitation) explicitly mentioned.”),

  “Weakness”: “Weakness mentioned (subjective or on exam).”,

  “Sensory”: “Sensory loss/paresthesias/numbness/tingling by exam or history.”,

  “Ataxia”: “Ataxia/incoordination/dysmetria.”,

  “Tremor”: “Tremor explicitly mentioned.”,

  “Bladder”: “Bladder symptoms (incontinence, urgency, retention, frequency).”,

  “Bowel”: “Bowel symptoms (constipation, diarrhea, incontinence) explicitly mentioned.”,

  “Cognitive”: “Memory/cognitive impairment/brain fog explicitly mentioned.”,

  “Falls”: “Falls explicitly mentioned.”,

  “Fatigue”: “Fatigue explicitly mentioned.”,

  “Gait”: “Gait/balance difficulty; assistive device use (cane/walker/wheelchair).”,

  “Pain”: (

    “Pain explicitly mentioned, including painful cramps, ”

    “burning pain, or~neuropathic pain.”)

  }

## Appendix D. Ancillary Tables

**Table A1:** Macro-averaged global performance against the adjudicated reference annotation set. Values are point estimates with note-level bootstrap 95% confidence intervals in parentheses. Macro-averaged metrics are unweighted means across the 17 phenotype categories.

Method	Macro Precision	Macro Recall	Macro F1
Annotator 2	0.92 (0.85–0.96)	0.78 (0.70–0.84)	0.83 (0.75–0.87)
Annotator 1	0.78 (0.72–0.82)	0.80 (0.73–0.85)	0.78 (0.72–0.81)
GPT-5.2	0.73 (0.67–0.80)	0.92 (0.86–0.95)	0.80 (0.74–0.84)
Llama-3.1 8B	0.60 (0.53–0.66)	0.85 (0.78–0.90)	0.69 (0.62–0.73)
ClinPhen	0.72 (0.64–0.78)	0.59 (0.50–0.66)	0.62 (0.53–0.67)
Doc2Hpo	0.56 (0.46–0.62)	0.69 (0.56–0.75)	0.59 (0.48–0.63)
PhenoSnap	0.55 (0.49–0.62)	0.76 (0.69–0.82)	0.61 (0.54–0.65)
BioClinical ModernBERT	0.16 (0.12–0.19)	0.24 (0.21–0.26)	0.17 (0.15–0.20)

**Table A2:** Micro-averaged global performance against the adjudicated reference annotation set. Values are point estimates with note-level bootstrap 95% confidence intervals in parentheses. Micro-averaged metrics are pooled across all note–category decisions.

Method	Micro Precision	Micro Recall	Micro F1
Annotator 2	0.94 (0.90–0.97)	0.80 (0.75–0.85)	0.86 (0.83–0.90)
Annotator 1	0.84 (0.80–0.89)	0.83 (0.78–0.88)	0.83 (0.80–0.87)
GPT-5.2	0.76 (0.71–0.81)	0.92 (0.89–0.95)	0.83 (0.80–0.87)
Llama-3.1 8B	0.64 (0.58–0.69)	0.85 (0.81–0.89)	0.73 (0.69–0.77)
ClinPhen	0.75 (0.69–0.81)	0.57 (0.51–0.64)	0.65 (0.60–0.70)
Doc2Hpo	0.59 (0.50–0.68)	0.62 (0.55–0.68)	0.60 (0.54–0.66)
PhenoSnap	0.55 (0.50–0.61)	0.71 (0.67–0.76)	0.62 (0.58–0.66)
BioClinical ModernBERT	0.43 (0.37–0.49)	0.50 (0.44–0.55)	0.46 (0.41–0.51)

**Table A3:** Pooled Matthews correlation coefficient (MCC) for each annotation method relative to the adjudicated reference annotation set. MCC was computed over all pooled note–category decisions. Values in parentheses are 95% confidence intervals estimated by note-level bootstrap resampling. ClinPhen, Doc2Hpo, and PhenoSnap were not able to process all notes, yielding totals less than 100. The reasons for processing failure were not investigated.

Method	Available Notes	Pooled MCC
Annotator 2	100	0.84 (0.80–0.88)
Annotator 1	100	0.80 (0.76–0.84)
GPT-5.2	100	0.80 (0.76–0.84)
Llama-3.1 8B	100	0.68 (0.63–0.72)
ClinPhen	73	0.59 (0.52–0.64)
Doc2Hpo	63	0.52 (0.45–0.59)
PhenoSnap	86	0.52 (0.48–0.57)
BioClinical ModernBERT	100	0.35 (0.29–0.41)
